# Supplementary material for: Genome-wide screen identifies novel factors for surface protein cross-wall trafficking and cell envelope homeostasis in Staphylococcus aureus
Source: J Bacteriol. 2026 Mar 19;208(4):e00523-25. doi: 10.1128/jb.00523-25 (PMC13104630; doi:10.1128/jb.00523-25)
Supplement: Supplemental figures and tables — Figures S1 to S3 and Tables S1 to S6. [file jb.00523-25-s0001.docx]

**Supplemental materials**


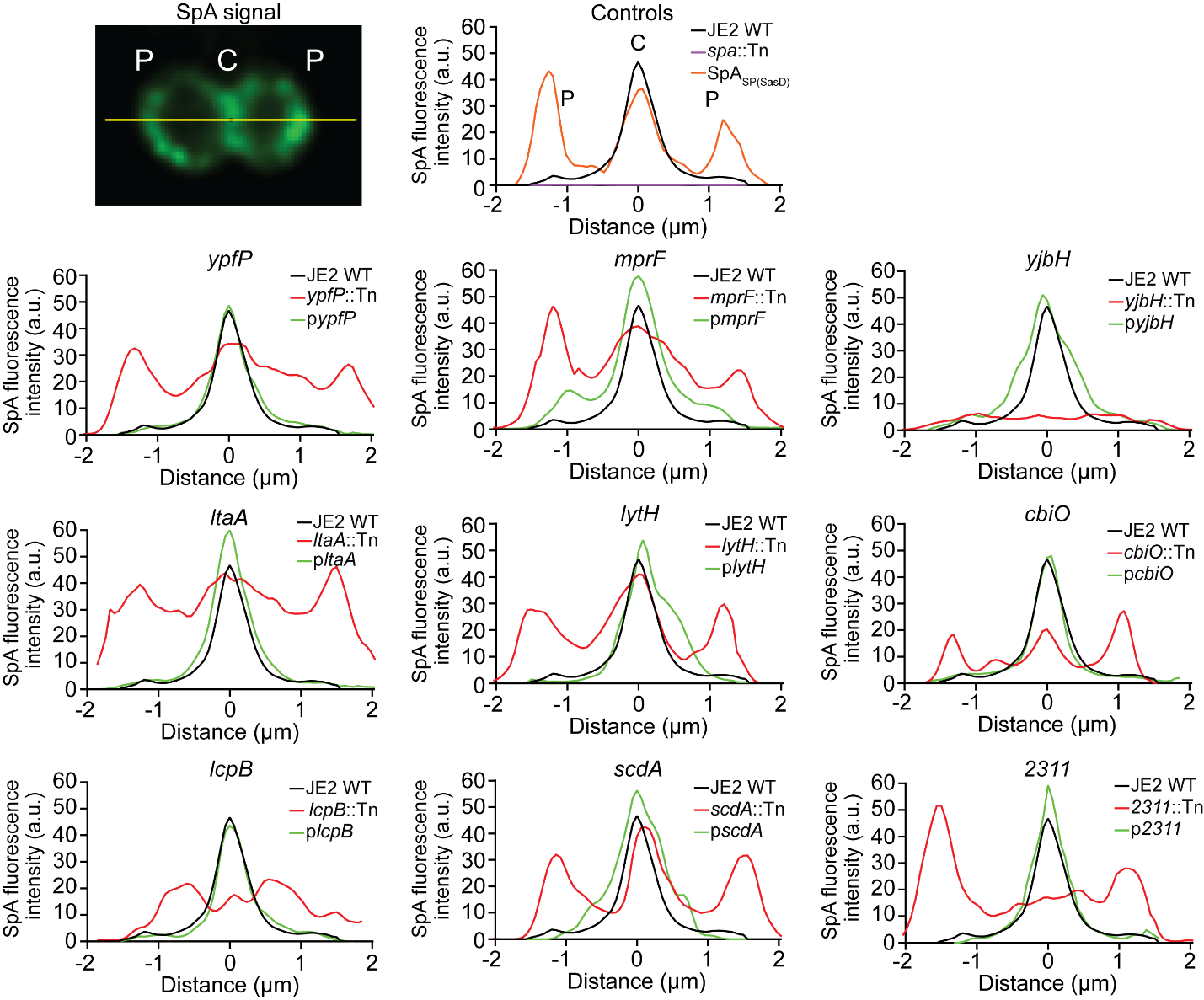


**Fig. S1.** Quantification of SpA fluorescence intensity at cross-wall versus peripheral wall. Histogram plotting SpA fluorescence signal along a line perpendicular to the cross-wall, example image shown in upper left. The peak at distance ‘0’ represents signals at the cross-wall site (C), and the two shoulders at distance ‘-1’ and ‘1’ represent signals at peripheral wall (P). Arbitrary units (a.u.) for fluorescence intensity are used on y-axis.


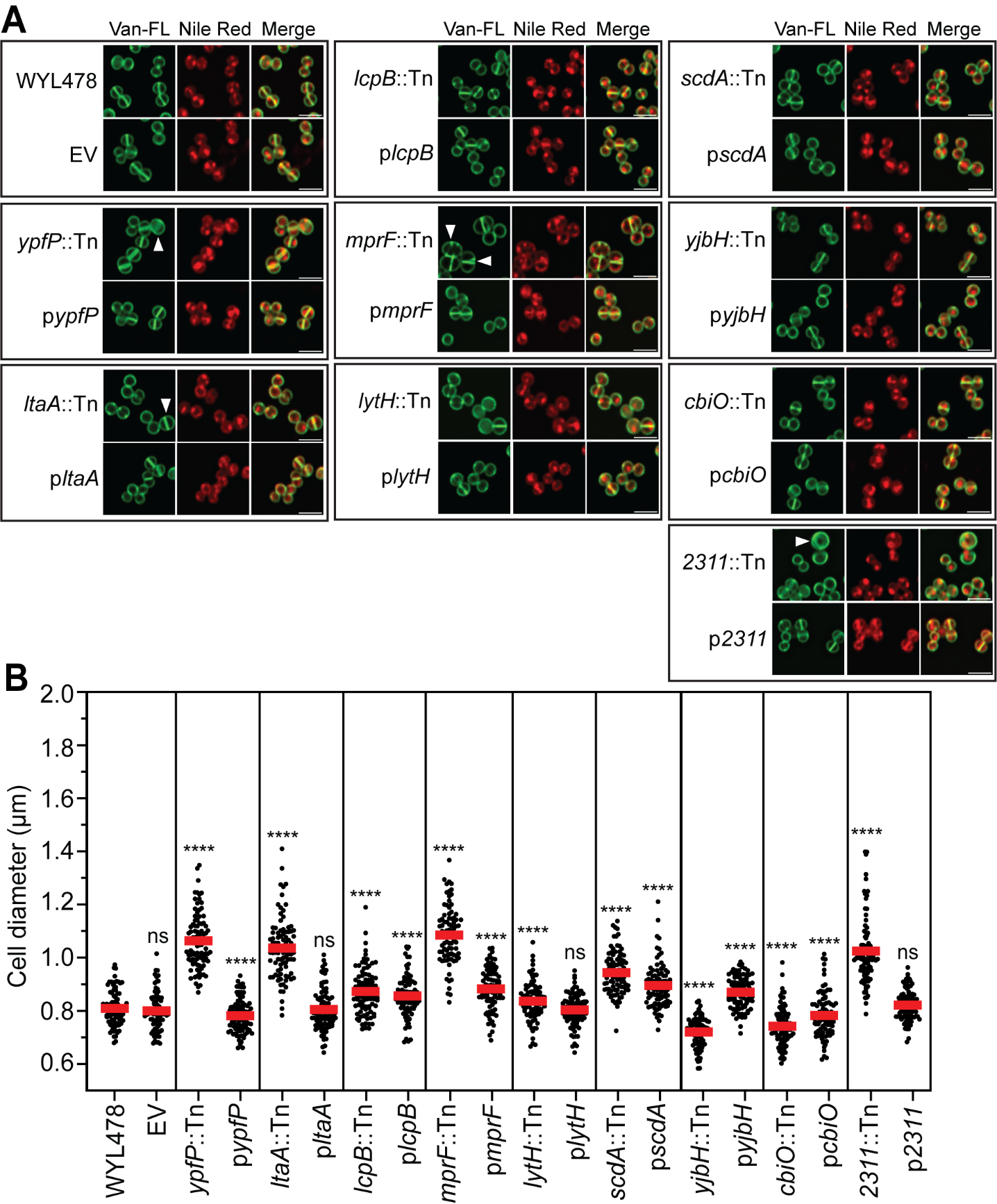


**Fig. S2.** Fluorescent vancomycin (Van-FL) labeling of WYL478 transductant mutants. A) Fluorescence images showing cell morphology. Van-FL stains cell wall and Nile red stains membrane. White arrowheads indicate cells with aberrant morphology compared to WYL478. B) Quantification of cell diameter based on the images represented in panel A, n = 90 cells. Unpaired *t*-test with Welch’s correction was performed for statistical analysis: **P* < 0.05; ***P* < 0.005; ****P* < 0.0005; *****P* < 0.0001. The exact mean values, standard deviation and *p* values are listed in Table S4.

**
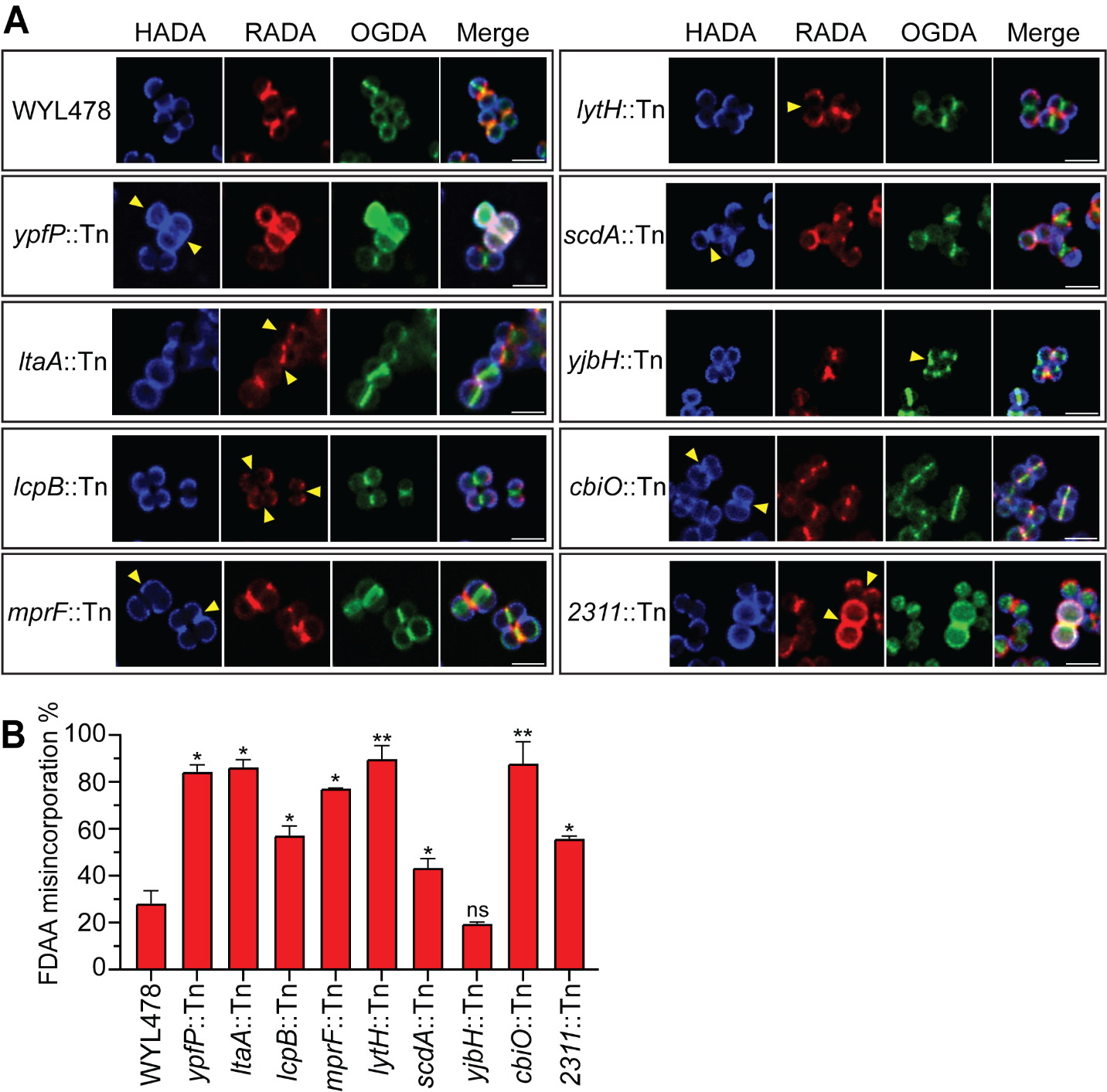
**

**Fig. S3.** Sequential FDAAs labeling of WYL478 transductant mutants. A) Fluorescence images of cells labeled with FDAAs: HADA (blue, first), RADA (red, second) and OGDA (green, third), yellow arrows indicate aberrant FDAA localization. B) Quantification of FDAA misincorporation. Unpaired t-test with Welch’s correction was performed for statistical analysis: **p* < 0.05, ***p* < 0.005. Representative images and quantification are from three independent experiments.

**Table S1.** Strains and plasmids used in this study.

| **Strain or plasmid** | **Description** | **Reference or source** |
| --- | --- | --- |
| *E. coli* |  |  |
| DH5α | Plasmid shuttle strain |  |
| DH5αλ*pir* | Cloning strain for pKK30 constructs | (1) |
| *S. aureus* |  |  |
| USA300 JE2 | Wild type background for NTML | (2) |
| JE2 *spa*::Tn | Transposon mutant of protein A (*spa*) | (2) |
| JE2 *cbiO*::Tn | Transposon mutant of *cbiO* | (2) |
| JE2 *lcpB*::Tn | Transposon mutant of *lcpB* | (2) |
| JE2 *ltaA*::Tn | Transposon mutant of *ltaA* | (2) |
| JE2 *lytH*::Tn | Transposon mutant of *lytH* | (2) |
| JE2 *mprF*::Tn | Transposon mutant of *mprF* | (2) |
| JE2 *2311*::Tn | Transposon mutant of *2311* | (2) |
| JE2 *scdA*::Tn | Transposon mutant of *scdA* | (2) |
| JE2 *yjbH*::Tn | Transposon mutant of *yjbH* | (2) |
| JE2 *ypfP*::Tn | Transposon mutant of *ypfP* | (2) |
| JE2 ∆*tagO*::*ermB* | Deletion mutant of *tagO* | This study |
| JE2 ∆*tagO*::*ermB* pRB473-*tagO* | Complementation strain for *tagO* mutant (*tagO* comp) | This study |
| WYL478 | SEJ1 pCL55*_itet_*-*spa*, RN4220∆spa complemented with ATc-inducible *spa* | (3) |
| WYL480 | SEJ1 pCL55*_itet_*, RN4220∆*spa* with empty vector (EV) | (3) |
| WYL478 *cbiO*::Tn | Transposon mutant of *cbiO* | This study |
| WYL478 *lcpB*::Tn | Transposon mutant of *lcpB* | This study |
| WYL478 *ltaA*::Tn | Transposon mutant of *ltaA* | This study |
| WYL478 *lytH*::Tn | Transposon mutant of *lytH* | This study |
| WYL478 *mprF*::Tn | Transposon mutant of *mprF* | This study |
| WYL478 *2311*::Tn | Transposon mutant of *2311* | This study |
| WYL478 *scdA*::Tn | Transposon mutant of *scdA* | This study |
| WYL478 *yjbH*::Tn | Transposon mutant of *yjbH* | This study |
| WYL478 *ypfP*::Tn | Transposon mutant of *ypfP* | This study |
| pKK30 EV | Vector used for complement construction | (1) |
| pKK30-P*_gdpP_*-*cbiO* | Complementation construct for *cbiO*::Tn mutants | This study |
| pKK30-P*_gdpP_*-*lcpB* | Complementation construct for *lcpB*::Tn mutants | This study |
| pKK30-P*_gdpP_*-*ltaA* | Complementation construct for *ltaA*::Tn mutants | This study |
| pKK30-P*_gdpP_*-*lytH* | Complementation construct for *lytH*::Tn mutants | This study |
| pKK30-P*_gdpP_*-*mprF* | Complementation construct for *mprF*::Tn mutants | This study |
| pKK30-P*_2310/2311_*-*2310/2311* | Complementation construct for *2311*::Tn mutants | This study |
| pKK30-P*_gdpP_*-*scdA* | Complementation construct for *scdA*::Tn mutants | This study |
| pKK30-P*_gdpP_*-*yjbH* | Complementation construct for *yjbH*::Tn mutants | This study |
| pKK30-P*_gdpP_*-*ypfP* | Complementation construct for *ypfP*::Tn mutants | This study |

**Table S2.** Primer list.

| **Primer name** | **Sequence** |
| --- | --- |
| 532-0903, yjbH, F | tctcccatcccctttgtttagcgtcgt |
| 533-0903, yjbH, R | tggtgcattaacgacatatcgccaaga |
| 534-0924, F | aagtgttgttgggaaagtgcttggt |
| 535-0924, R | ttgtacgaataccgccaccaaccga |
| 536-2176, F | tccatcaacagacgatgggcatagt |
| 537-2176, R | acactcgtatccttaaacaaggccctcgtc |
| 538-0438, F | gcgttgcaggaagatgtcaaagatgga |
| 539-0438, R | ccacccgggaaataatacttttcgcga |
| 540-1531, F | cccctaaaacacgtggtatatcaag |
| 541-1531, R | gtgcctaaagcagaaccggtggagc |
| 542-1336, F | ccgtgtgggacgtcatcatgtttaac |
| 543-1336, R | gggattagaagctgttgttgctaggg |
| 544-0988, F | acccagtttgggaagttacgaatg |
| 545-0988, R | caggggccccaacatagagaatttc |
| 546-2127, F | ctccaaaattcggagctaaggctgc |
| 547-2127, R | cctgtggttgaatttaatcgttcg |
| 548-0752, F | accgactcgttaattgtgaaacggcca |
| 549-0752, R | tgggcaccaagtcttgcaatgcgtct |
| 550-0657, F | agtaggttgtatgtgtcaaccggca |
| 551-0657, R | tggcaagattgtaacacctacacca |
| 552-0658, F | tcctttagaggtgacggtagttgga |
| 553-0658, R | tgggaccgtaaccgcaatacccata |
| 554-0017, F | tctggttcgattgagcaagatgccga |
| 555-0017, R | tctgtccaacctggtaactcttca |
| 556-0238, F | tgttacaggtaagcctgccaatgct |
| 557-0238, R | tccttatcaatgtcagccgattcct |
| 558-0917, F | tggccctatcattgagccgaatcct |
| 559-0917, R | acagccgcccagataaactagaca |
| 560-0918, F | tgaaggcaaagcgtgttgggtgcact |
| 561-0918, R | acggagggtggctattaatggttact |
| 587_2044, F | accctattcgacatacctttactac |
| 588_2044, R | tctgttcccattcttcgtcaggacc |
| 589_0739, F | accgaggctctaaaagggcgtcggt |
| 590_0739, R | agcaactgaaagtatttggccttct |
| 591_0651, F | tggagcactgcgcaacgaatgatca |
| 592_0651, R | Tcctccttctaccgtcgttttatgcg |
| 593_2504, F | tgccccatttgtaccaagctctact |
| 594_2504, R | ggtttacattagaggataggctgca |
| 595_2356, F | tgatcaacacgatcaccgttgcctt |
| 596_2356, R | tggcgatgaagccatatacatgatg |
| 597_1642, F | tgctgtcattggatgcacgtgatct |
| 598_1642, R | aaagattgcaacatgtggaacgcca |
| 599_2282, F | tgcgccccttgtttaaagcaacacc |
| 600_2282, R | ttattgtaggcactcaagagggtgt |
| 615_0270, F | tggattgccaggtggatttacttca |
| 616_0270, R | accgatgccaccagacatacgttgga |
| 617_2253, F | acctatttaaacgccgacgtccacca |
| 618_2253, R | aagcgcgtacttttgatggaggtga |
| 619_1720, F | atgtcctgccccacttgcattggt |
| 620_1720, R | tcgggaggtaacaatggattacgca |
| 621_1509, F | ccaatgaatcctccaatatgcgcca |
| 622_1509, R | agcttgtacggaatgtggtgacaga |
| 626_PgdpP_F | GGGGCTAGCtaatatatattttcaaagaatgaagcca |
| 627_PgdpP_R | GGGGAGCTCatttttaactatagctattattttaacct |
| 630_00953, F | GGGGCTAGCtttattatacacgttttgttgatgt |
| 631_00953, R | GGGGAGCTCttatttaacgaagaatcttgcatat |
| 632_Pkk30 F | aacgtatcttatttaaagtgcgttg |
| 633_Pkk30 R | agcggtgattgtttaccatcaattt |
| 655 mprF pKK30 F | GGGGCTAGCgtaataaaaaataccaatgactatatgt |
| 656_mprF pKK30 R | CCCGGATCCtaattatttgtgacgtattacacgca |
| 664R_pKK30_seq | accatcaattttaacaatcaccgctact |
| 665_ltaA. F | GGGGAGCTCaccacaagagatttatgga |
| 666_ltaA, R | CCCGGATCCttacttagctttttctctatttact |
| 667_lytTR, F | GGGGAGCTCaaggagcgcttatcaatatga |
| 668_lytTR, R | GGGGGATCCtcattttaattgtttcagttgcttgt |

**Table S3.** Quantification of SpA localization in JE2 and WYL478 background.

| **Strain** | **Avg. % SpA at cross-wall** | **Avg. % SpA at peripheral wall** | **Avg. No SpA signal** | **CW p-value** | **PW p-value** | **No SpA p-value** |
| --- | --- | --- | --- | --- | --- | --- |
| JE2 WT | 33.86 ± 2.7 | 3.90 ± 1.3 | 62.23 ± 3.1 |  |  |  |
| *spa*::Tn | 0.0 ± 0 | 0.0 ± 0 | 100 ± 0 | 0.0020 | 0.0358 | 0.0022 |
| SpA_SP(SasD)_ | 1.50 ± 1.3 | 58.77 ± 8.3 | 39.73 ± 9.1 | 0.0004 | 0.0064 | 0.0394 |
| *ypfP*::Tn | 0.0 ± 0 | 5.74 ± 2.7 | 94.25 ± 2.7 | 0.0004 | <0.0001 | 0.0724 |
| p*ypfP* | 32.43 ± 4.3 | 3.00 ± 2.8 | 64.55 ± 3.6 | 0.5307 | 0.2689 | 0.2856 |
| *ltaA*::Tn | 0.99 ± 1.1 | 47.93 ± 6.7 | 51.07 ± 6.1 | 0.0098 | 0.0013 | 0.0498 |
| p*ltaA* | 28.48 ± 0.8 | 3.77 ± 3.3 | 67.74 ± 4.0 | 0.0302 | 0.8656 | 0.1287 |
| *lcpB*::Tn | 0.23 ± 0.4 | 10.03 ± 5.2 | 89.73 ± 5.6 | 0.0017 | 0.1737 | 0.0045 |
| p*lcpB* | 26.55 ± 5.5 | 3.09 ± 3.3 | 70.34 ± 8.8 | 0.1352 | 0.7231 | 0.2462 |
| *mprF*::Tn | 6.36 ± 1.4 | 37.18 ± 5.9 | 56.45 ± 5.6 | 0.0006 | 0.0060 | 0.0676 |
| p*mprF* | 33.48 ± 2.7 | 2.56 ± 2.3 | 63.95 ± 2.3 | 0.0615 | 0.9521 | 0.1344 |
| *lytH*::Tn | 3.15 ± 3.5 | 38.80 ± 9.5 | 58.05 ± 6.0 | 0.0004 | 0.0221 | 0.3608 |
| p*lytH* | 30.05 ± 5.2 | 1.47 ± 1.8 | 68.46 ± 23.6 | 0.3387 | 0.1359 | 0.0869 |
| *scdA*::Tn | 0.43 ± 0.7 | 0.36 ± 0.6 | 99.20 ± 1.4 | 0.0006 | 0.0078 | 0.2101 |
| p*scdA* | 32.32 ± 4.3 | 4.80 ± 3.2 | 62.87 ± 2.6 | 0.8734 | 0.4419 | 0.4868 |
| *yjbH*::Tn | 0.0 ± 0 | 6.91 ± 2.5 | 93.08 ± 2.5 | 0.0011 | 0.0268 | 0.0005 |
| p*yjbH* | 27.50 ± 7.8 | 1.29 ± 1.1 | 71.20 ± 7.9 | 0.6325 | 0.6848 | 0.7972 |
| *cbiO*::Tn | 4.16 ± 3.4 | 39.05 ± 1.7 | 56.78 ± 1.9 | 0.0020 | 0.1644 | 0.0002 |
| p*cbiO* | 32.55 ± 1.9 | 1.99 ± 2.1 | 65.44 ± 3.3 | 0.2894 | 0.0604 | 0.1776 |
| *2311*::Tn | 1.9 ± 0.8 | 27.81 ± 4.3 | 70.28 ± 3.5 | 0.0020 | 0.3701 | 0.0002 |
| p*2311* | 33.56 ± 3.7 | 1.51 ± 1.7 | 64.92 ± 3.0 | 0.6558 | 0.6494 | 0.4482 |
| **Strain** | **Avg. % SpA at cross-wall** | **Avg. % SpA at peripheral wall** | **Avg. No SpA signal** | **CW p-value** | **PW p-value** | **No SpA p-value** |
| WYL478 | 33.86 ± 1.4 | 6.55 ± 2.3 | 59.58 ± 1.6 |  |  |  |
| EV | 0.0 ± 0 | 0.0 ± 0 | 100 ± 0 | 0.0005 | 0.0405 | 0.0005 |
| SpA_SP(SasD)_ | 0.92 ± 1.6 | 43.32 ± 5.9 | 55.75 ± 6.9 | <0.0001 | 0.0036 | 0.4398 |
| *ypfP*::Tn | 1.61 ± 1.4 | 44.23 ± 6.3 | 54.15 ± 6.3 | <0.0001 | 0.0044 | 0.2735 |
| p*ypfP* | 27.94 ± 7.9 | 1.80 ± 1.6 | 70.24 ± 7.3 | 0.3249 | 0.0523 | 0.1210 |
| *ltaA*::Tn | 10.19 ± 6.2 | 44.23 ± 6.9 | 45.57 ± 7.3 | 0.0180 | 0.0062 | 0.0731 |
| p*ltaA* | 26.35 ± 8.9 | 4.73 ± 4.6 | 69.01 ± 8.5 | 0.2817 | 0.5843 | 0.1905 |
| *lcpB*::Tn | 3.13 ± 1.6 | 36.43 ± 7.1 | 60.43 ± 6.4 | <0.0001 | 0.0117 | 0.8410 |
| p*lcpB* | 25.77 ± 2.1 | 0.69 ± 1.2 | 73.52 ± 3.3 | 0.0083 | 0.0316 | 0.0082 |
| *mprF*::Tn | 0.0 ± 0 | 51.43 ± 3.6 | 48.56 ± 3.6 | 0.0005 | 0.0002 | 0.0208 |
| p*mprF* | 28.44 ± 7.4 | 3.54 ± 3.1 | 68.33 ± 8.1 | 0.3335 | 0.2596 | 0.1983 |
| *lytH*::Tn | 4.34 ± 2.3 | 51.83 ± 11.2 | 43.81 ± 9.5 | 0.0002 | 0.0165 | 0.0997 |
| p*lytH* | 20.17 ± 7.0 | 0.0 ± 0 | 79.82± 7.0 | 0.0724 | 0.0405 | 0.0323 |
| *scdA*::Tn | 3.73 ± 4.6 | 28.38 ± 12.6 | 67.88 ± 14.8 | 0.0045 | 0.0913 | 0.4336 |
| p*scdA* | 19.07 ± 3.6 | 1.40 ± 2.4 | 79.52 ± 2.5 | 0.0116 | 0.0577 | 0.0007 |
| *yjbH*::Tn | 0.0 ± 0 | 0.0 ± 0 | 100 ± 0 | 0.0005 | 0.0405 | 0.0005 |
| p*yjbH* | 28.86 ± 11.0 | 2.82 ± 2.5 | 68.34 ± 8.5 | 0.5139 | 0.1368 | 0.2140 |
| *cbiO*::Tn | 2.19 ± 0.2 | 41.36 ± 2.2 | 56.44 ± 2.2 | 0.0005 | <0.0001 | 0.1281 |
| p*cbiO* | 31.74 ± 15.1 | 6.35 ± 5.0 | 62.01 ± 16.7 | 0.8300 | 0.9562 | 0.8250 |
| *2311*::Tn | 3.18 ± 0.7 | 36.86 ± 2.3 | 59.96 ± 2.1 | <0.0001 | <0.0001 | 0.8184 |
| p*2311* | 26.65 ± 3.4 | 3.63 ± 2.2 | 69.92 ± 2.7 | 0.0534 | 0.1894 | 0.0084 |

**Table S4.** Quantification of cell diameter in JE2 and WYL478 background.

| **Strain** | **Average diameter (μm)** | **p-value** | **Strain** | **Average diameter (μm)** | **p-value** |
| --- | --- | --- | --- | --- | --- |
| JE2 WT | 0.848 ± 0.05 |  | WYL478 | 0.809 ± 0.03 |  |
| *spa*::Tn | 0.851 ± 0.04 | 0.8169 | EV | 0.799 ± 0.02 | 0.3846 |
| *ypfP*::Tn | 0.981 ± 0.04 | <0.0001 | *ypfP*::Tn | 1.064 ± 0.02 | <0.0001 |
| p*ypfP* | 0.852 ± 0.06 | 0.5048 | p*ypfP* | 0.781 ± 0.01 | 0.0044 |
| *ltaA*::Tn | 1.00 ± 0.04 | <0.0001 | *ltaA*::Tn | 1.034 ± 0.06 | <0.0001 |
| p*ltaA* | 0.864 ± 0.07 | 0.1559 | p*ltaA* | 0.806 ± 0.04 | 0.7095 |
| *lcpB*::Tn | 0.898 ± 0.01 | <0.0001 | *lcpB*::Tn | 0.873 ± 0.03 | <0.0001 |
| p*lcpB* | 0.856 ± 0.05 | 0.4241 | p*lcpB* | 0.854 ± 0.03 | <0.0001 |
| *mprF*::Tn | 1.00 ± 0.02 | <0.0001 | *mprF*::Tn | 1.085 ± 0.01 | <0.0001 |
| p*mprF* | 0.841 ± 0.04 | 0.6700 | p*mprF* | 0.881 ± 0.05 | <0.0001 |
| *lytH*::Tn | 0.841 ± 0.02 | 0.6969 | *lytH*::Tn | 0.836 ± 0.02 | 0.0080 |
| p*lytH* | 0.849 ± 0.02 | 0.8165 | p*lytH* | 0.803 ± 0.01 | 0.5529 |
| *scdA*::Tn | 0.902 ± 0.05 | <0.0001 | *scdA*::Tn | 0.943 ± 0.02 | <0.0001 |
| p*scdA* | 0.872 ± 0.01 | 0.0212 | p*scdA* | 0.894 ± 0.05 | <0.0001 |
| *yjbH*::Tn | 0.742 ± 0.02 | <0.0001 | *yjbH*::Tn | 0.720 ± 0.02 | <0.0001 |
| p*yjbH* | 0.880 ± 0.03 | 0.0010 | p*yjbH* | 0.869 ± 0.01 | <0.0001 |
| *cbiO*::Tn | 0.751 ± 0.01 | <0.0001 | *cbiO*::Tn | 0.741 ± 0.01 | <0.0001 |
| p*cbiO* | 0.851 ± 0.07 | 0.7749 | p*cbiO* | 0.781 ± 0.03 | 0.0171 |
| *2311*::Tn | 1.09 ± 0.03 | <0.0001 | *2311*::Tn | 1.025 ± 0.01 | <0.0001 |
| p*2311* | 0.859 ± 0.01 | 0.2235 | p*2311* | 0.822 ± 0.02 | 0.1743 |

**Table** **S5.** Quantification of cell cycle analysis in JE2 background.

| **Strain** | **phase 1** | **phase 2** | **phase 3** | **phase 1 p-value** | **phase 2 p-value** | **phase 3 p-value** |
| --- | --- | --- | --- | --- | --- | --- |
| JE2 WT | 67.63 ± 1.34 | 13.12 ± 0.61 | 19.25 ± 0.92 |  |  |  |
| *ypfP*::Tn | 68.01 ± 1.07 | 21.98 ± 2.85 | 20.03 ± 1.52 | 0.0019 | 0.0282 | 0.4995 |
| p*ypfP* | 57.99 ± 1.71 | 13.59 ± 1.41 | 18.39 ± 2.01 | 0.7178 | 0.6329 | 0.5520 |
| *ltaA*::Tn | 73.42 ± 4.41 | 29.71 ± 3.10 | 12.95 ± 1.06 | 0.0307 | 0.0205 | 0.0010 |
| p*ltaA* | 57.34 ± 3.65 | 10.88 ± 1.75 | 15.70 ± 2.95 | 0.1415 | 0.1465 | 0.1637 |
| *lcpB*::Tn | 72.22 ± 4.27 | 18.32 ± 2.20 | 12.66 ± 3.03 | 0.6568 | 0.0464 | 0.0535 |
| p*lcpB* | 69.02 ± 4.59 | 12.12 ± 1.89 | 15.66 ± 2.75 | 0.1967 | 0.4625 | 0.1417 |
| *mprF*::Tn | 65.68 ± 1.99 | 23.36 ± 2.78 | 14.39 ± 2.22 | 0.0071 | 0.0197 | 0.0474 |
| p*mprF* | 62.25 ± 0.83 | 11.69 ± 0.96 | 22.36 ± 1.26 | 0.2420 | 0.1660 | 0.0298 |
| *lytH*::Tn | 66.87 ± 2.25 | 18.82 ± 3.35 | 16.24 ± 2.21 | 0.0565 | 0.0045 | 0.1017 |
| p*lytH* | 64.93 ± 3.10 | 13.79 ± 2.73 | 19.33 ± 2.40 | 0.6489 | 0.7128 | 0.9617 |
| *scdA*::Tn | 69.20 ± 3.24 | 28.78 ± 2.01 | 17.16 ± 4.12 | 0.0451 | 0.0030 | 0.4739 |
| p*scdA* | 54.06 ± 5.56 | 12.65 ± 2.47 | 18.15 ± 1.85 | 0.5001 | 0.7758 | 0.4253 |
| *yjbH*::Tn | 67.53 ± 3.78 | 25.21 ± 5.53 | 12.73 ± 2.59 | 0.0042 | 0.0002 | 0.0431 |
| p*yjbH* | 62.06 ± 3.78 | 13.37 ± 3.03 | 19.10 ± 1.11 | 0.9693 | 0.8995 | 0.8625 |
| *cbiO*::Tn | 67.46 ± 1.65 | 20.28 ± 4.29 | 16.95 ± 2.54 | 0.1970 | 0.0249 | 0.2622 |
| p*cbiO* | 62.77 ± 6.80 | 12.73 ± 2.60 | 19.81 ± 1.70 | 0.8985 | 0.8235 | 0.6533 |
| *2311*::Tn | 55.00 ± 7.50 | 27.77 ± 7.09 | 17.23 ± 3.62 | 0.0011 | 0.0082 | 0.1005 |
| p*2311* | 69.85 ± 4.80 | 11.81 ± 1.68 | 18.34 ± 3.20 | 0.5114 | 0.3102 | 0.6760 |

**Table S6.** Quantification of SpA localization and cell diameter of the ∆*tagO* mutant.

| **Strain** | **Avg. % SpA at cross-wall** | **Avg. % SpA at peripheral wall** | **Avg. No SpA signal** | **Average diameter (μm)** |
| --- | --- | --- | --- | --- |
| JE2 WT | 32.25 ± 2.1 | 3.04 ± 2.7 | 64.71 ± 4.1 | 0.795 ± 0.06 |
| ∆*tagO* | 0.0 ± 0.0 | 19.27 ± 1.2 | 80.72 ± 1.2 | 1.048 ± 0.18 |
| ∆*tagO* comp | 31.45 ± 0.9 | 16.62 ± 1.8 | 51.93 ± 2.7 | 0.838 ± 0.07 |
| *spa*::Tn | 0.0 ± 0.0 | 0.0 ± 0.0 | 0.0 ± 0.0 | 0.792 ± 0.08 |
| **Strain** | **CW p-value** | **PW p-value** | **No SpA p-value** | **Diameter p-value** |
| JE2 WT |  |  |  |  |
| ∆*tagO* | 0.0014 | 0.0035 | 0.0158 | <0.0001 |
| ∆*tagO* comp | 0.6095 | 0.0068 | 0.0262 | <0.0001 |
| *spa*::Tn | 0.0014 | 0.1931 | 0.0003 | 0.0046 |

1. Krute CN, Krausz KL, Markiewicz MA, Joyner JA, Pokhrel S, Hall PR, Bose JL. 2016. Generation of a Stable Plasmid for In Vitro and In Vivo Studies of Staphylococcus Species. Appl Environ Microbiol 82:6859-6869.

2. Fey PD, Endres JL, Yajjala VK, Widhelm TJ, Boissy RJ, Bose JL, Bayles KW. 2013. A genetic resource for rapid and comprehensive phenotype screening of nonessential Staphylococcus aureus genes. mBio 4:e00537-12.

3. Zhang R, Shebes MA, Kho K, Scaffidi SJ, Meredith TC, Yu W. 2021. Spatial regulation of protein A in Staphylococcus aureus. Mol Microbiol 116:589-605.
